# Supplementary material for: Bayesian refinement of protein structures and ensembles against SAXS data using molecular dynamics
Source: PLoS Comput Biol. 2017 Oct 18;13(10):e1005800. doi: 10.1371/journal.pcbi.1005800 (PMC5662244; doi:10.1371/journal.pcbi.1005800)
Supplement: S1 Archive — (BZ2) [file pcbi.1005800.s011.tar.bz2 › gromacs_4.62_bayesian_saxs_refi/share/html/online/g_options.html]

g\_options


| g\_options Main Table of Contents |

**VERSION 4.6  
Sat 19 Jan 2013**

---

### Description

GROMACS programs have some standard options,
of which some are hidden by default:

### Other options

| option | type | default | description |
| --- | --- | --- | --- |
| **-[no]h** | bool | no | Print help info and quit || **-[no]version** | bool | no | Print version info and quit || **-nice** | int | 0 | Set the nicelevel |

### Known problems

- If the configuration script found Motif or Lesstif on your system, you can use the graphical interface (if not, you will get an error):  
  -X gmx\_bool no Use dialog box GUI to edit command line options- When compiled on an SGI-IRIX system, all GROMACS programs have an additional option:  
    -npri int 0 Set non blocking priority (try 128)- Optional files are not used unless the option is set, in contrast to non-optional files, where the default file name is used when the option is not set.- All GROMACS programs will accept file options without a file extension or filename being specified. In such cases the default filenames will be used. With multiple input file types, such as generic structure format, the directory will be searched for files of each type with the supplied or default name. When no such file is found, or with output files the first file type will be used.- All GROMACS programs with the exception of mdrun and eneconv check if the command line options are valid. If this is not the case, the program will be halted.- Enumerated options (enum) should be used with one of the arguments listed in the option description, the argument may be abbreviated. The first match to the shortest argument in the list will be selected.- Vector options can be used with 1 or 3 parameters. When only one parameter is supplied the two others are also set to this value.- All GROMACS programs can read compressed or g-zipped files. There might be a problem with reading compressed .xtc, .trr and .trj files, but these will not compress very well anyway.- Most GROMACS programs can process a trajectory with fewer atoms than the run input or structure file, but only if the trajectory consists of the first n atoms of the run input or structure file.- Many GROMACS programs will accept the -tu option to set the time units to use in output files (e.g. for xmgr graphs or xpm matrices) and in all time options.

---

http://www.gromacs.org  
gromacs@gromacs.org
